# Abridged Therapeutics, Founded upon Histology and Cellular Pathology

**Published:** 1887-04

**Authors:** 


ABRIDGED THERAPEUTICS, FOUNDED UPON HISTOLOGY AND CELLULAR PATHOLOGY. By Dr. Med. Schüssler, of Oldenburg. Authorized translation by M. Docetti Walker. Baltimore: E. H. Holbrook, M. D.

This book is Dr. Schüssler's famous treatise on the twelve tissue remedies, but the copy before us is very much enlarged and improved since we first saw it ten or twelve years ago.

In support of the method of practice maintained in this book the author lays down the following propositions:

That the blood contains certain mineral salts in addition to water, fat, and albuminous substances:

That "in the formation of the tissue-cells these salts absolutely determine the kind of cell:"

That "a disturbance in the molecular movements of any of the inorganic salts of a tissue produces a disease:"

That "for the healing or cure of such, the smallest dose of the identical inorganic substance suffices, because the molecules of that substance administered as medicine fill up the gap in the chain of molecules of that particular cell or tissue-salt:"

He recommends only minute doses of these tissue salts, and offers an explanation of the value of (say) common table salt in minute doses when the patient is already taking salt in large quantities with his food.

The explanation is as follows:

"Through irritation, over-stimulation, a certain tissue has lost its molecules of common salt. In consequence of this, that portion of tissue is so changed that it is no longer able to absorb out of the plasma new molecules of salt. The requisite molecules must, therefore, be introduced by some other means. The molecules of a minimum dose of common salt given as medicine reach the neurilemma (nerve sheaths) of those branches of the sympathetic which ramify through the mucous membrane of the mouth and upper part of throat, etc.

"In this way they proceed to the nearest ganglia (nerve centres) and from there they pass by the same path, *i. e.*, the ducts of the connective tissue sheaths of other branches of the sympathetic into the diseased tissue. * * *

"When the said portion of tissue has acquired its former healthy condition through this supply of molecules, it possesses again the capability of absorbing *from the plasma* particles of common salt or any other cell salt."

"The presence of a dose of common salt unattenuated can be perceived by the nerves of taste. To produce this it is only necessary that the ends of these nerves be touched by the common salt. It is, however, questionable if the salt in a crude, non-attenuated condition can enter, or can be taken up by the ducts of the neurilemma which envelop the branches of the sympathetic.

"It seems probable that these narrow canals can only take up the delicately fine attenuated molecules of Sodium chloride and the other tissue salts, when set free by a special process of trituration or subdivision."

In the selection of the above extracts we have endeavored to give the readers of this journal who may never have seen the book itself in any of its editions, the salient features of a system that has attracted considerable attention in our school, and has sometimes been denominated "Schüsslerism."

For ourselves, as we understand Homœopathy, we cannot approve of these theories. We do not see how it is possible for them to be true. To us this whole scheme seems like a patching together of the theories and facts of physiology and the principles of Homœopathy in a vain endeavor to reconcile them.

The treatment of the sick is made to depend upon theories and "views." This has been the fate of sick people in all ages of the world under ordinary medicine. As these "views" were perpetually changing, the patient was subjected to a continual variation in therapeutic measures, and under such uncertainty, of course, the diseased state was seldom relieved, while the endurance of the patient was still further taxed.

The system under consideration seems to us to be only a repetition of the old story—a new theory, rising, flourishing, and then declining, until it sinks into oblivion. It apparently aims at establishing routine in medical practice, and thus to relieve the physician of the exertion and loss of time required for original thought in individualizing each case. Its ultimate end is to revive the idea and the search for specifics; to treat diseases rather than sick individuals, and to exempt the medical mind from the drudgery of study. All these are—if we may be allowed to use such an expression—the *vices* of old medicine. Under their influence medicine has languished. Homœopathy was supposed to cure them, but in this Schüssler system we find them revived, and Homœopathy ingeniously made to support them.

Dr. Schüssler has stated some of the objections to his system and refuted them in a special chapter. But to us these objections seem puerile and, as a consequence, the "refutations" valueless. The writers making the objections do not seem to have made them from the point of view of a profound knowledge of and conviction of the law of the similars. Neither they nor the author of the book realize the comprehensive significance of this law. It is doubtful if they have sufficient acquaintance with it to see how it could be a law. Certain it is that there is not one objection that can be attributed to homœopathic conviction.

Perhaps it will be said that this criticism is severe and unjust. It is not our desire to be unjust, but we are anxious for the truth. We are free to say that the theory excites our admiration, but it is *only* a theory. If it is backed up by some clinical cases, we must say that they are not enough to establish it. Such a small number of cases would not be sufficient to establish the truth of Homœopathy. But, after all, the objections to it are cardinal and have been already stated.

Dismissing this part of the subject and returning to the make-up of the book, we can assure our readers that it is well printed; that it is well arranged for finding the most suitable remedy for any given case, and that it has that most desirable addition to a book—a copious index. W. M. J.

